# Biological drivers of clinical phenotype in myelofibrosis

**DOI:** 10.1038/s41375-022-01767-y

**Published:** 2022-11-24

**Authors:** John Mascarenhas, Hélène F. E. Gleitz, Helen T. Chifotides, Claire N. Harrison, Srdan Verstovsek, Alessandro Maria Vannucchi, Raajit K. Rampal, Jean-Jacques Kiladjian, William Vainchenker, Ronald Hoffman, Rebekka K. Schneider, Alan F. List

**Affiliations:** 1 Tisch Cancer Institute, Icahn School of Medicine at Mount Sinai, New York, NY USA; 2 Department of Developmental Biology, Erasmus Medical Center, Rotterdam, The Netherlands; 3 Oncode Institute, Erasmus Medical Center, Rotterdam, The Netherlands; 4 Leukemia Department, The University of Texas MD Anderson Cancer Center, Houston, TX USA; 5 Guy’s and St Thomas’ NHS Foundation Trust, London, UK; 6 Azienda Ospedaliero-Universitaria Careggi, Firenze, Italy; 7 Leukemia Service, Department of Medicine and Center for Hematologic Malignancies, Memorial Sloan Kettering Cancer Center, New York, NY 10065 USA; 8 Hôpital Saint-Louis, AP-HP, Université Paris Cité, Paris, France; 9 Gustave Roussy, INSERM UMR1287, Université Paris-Saclay, Villejuif, France; 10 Institute of Cell and Tumor Biology, RWTH Aachen University, Medical Faculty, Aachen, Germany; 11 Precision BioSciences, Inc, Durham, NC USA

**Keywords:** Myeloproliferative disease, Myeloproliferative disease

## Abstract

Myelofibrosis (MF) is a myeloproliferative disorder that exhibits considerable biological and clinical heterogeneity. At the two ends of the disease spectrum are the myelodepletive or cytopenic phenotype and the myeloproliferative phenotype. The cytopenic phenotype has a high prevalence in primary MF (PMF) and is characterized by low blood counts. The myeloproliferative phenotype is typically associated with secondary MF (SMF), mild anemia, minimal need for transfusion support, and normal to mild thrombocytopenia. Differences in somatic driver mutations and allelic burden, as well as the acquisition of non-driver mutations further influences these phenotypic differences, prognosis, and response to therapies such as JAK2 inhibitors. The outcome of patients with the cytopenic phenotype are comparatively worse and frequently pose a challenge to treat given the inherent exacerbation of cytopenias. Recent data indicate that an innate immune deregulated state that hinges on the myddosome-IRAK-NFκB axis favors the cytopenic myelofibrosis phenotype and offers opportunity for novel treatment approaches. We will review the biological and clinical features of the MF disease spectrum and associated treatment considerations.

## Introduction

The *BCR-ABL1-*negative myeloproliferative neoplasms (MPNs) are a group of chronic hematopoietic stem and progenitor cell (HSPC) derived hematologic malignancies, which include essential thrombocythemia (ET), polycythemia vera (PV) and primary myelofibrosis (PMF). ET and PV can both progress to a form of secondary myelofibrosis (sMF) that collectively with PMF are simply termed myelofibrosis (MF). Hyperactivity of the JAK-STAT signaling pathway is the central biologic hallmark of these diseases and somatic mutations involving the *JAK2, CALR* and *MPL* genes comprise 90% of driver mutations [1]. Additionally, non-driver mutations frequently occur in genes involving signal transduction, epigenetic modifiers, spliceosome and tumor suppressor pathways that further influence phenotype and prognosis [2]. MF is characterized at a biological level by expansion of a malignant HSPC with aberrant trafficking to extramedullary sites of hematopoiesis [3]. The histopathological consequences are typified by bone marrow hypercellularity, reticulin and collagen fibrosis, and a high frequency of circulating CD34 + cells. The clinical picture is heterogeneous but in general includes progressive cytopenias, organomegaly, debilitating systemic symptoms, and potential for evolution to acute myeloid leukemia. The incidence of MF is 0.44 per 100,000 person-years, with a median age at time of diagnosis of approximately 68 years and a median survival of 5.2–5.9 years [4–7]. Given the highly variable clinical presentation, prognostic scales have been developed to guide treatment decisions.

### Prognostication in myelofibrosis

Prognostication for MF has evolved over the years and originated with the Lille classification which included leukocytosis or leukopenia and anemia to define three prognostic risk categories (Table 1) [8]. The International Prognostic Scoring System (IPSS) which is applied at the time of diagnosis includes advanced age, leukocytosis, anemia, systemic symptoms, and peripheral blood blasts to create four prognostic categories [6]. The Dynamic IPSS (DIPSS) utilizes the same risk variables but can be applied at any point in the disease course [9]. Subsequently, the DIPSS-plus incorporated thrombocytopenia (<100 ×10^9^/L), adverse karyotype, and red blood cell transfusion dependence to predict overall survival (OS) and determined that adverse karyotype or thrombocytopenia also predict leukemia free survival (LFS) [10]. The modern Mutation-enhanced International Prognostic Scoring System (MIPSS) and Genetically Inspired Prognosis Scoring System (GIPSS) further refine prognostication by integrating cytogenetic and molecular data [11, 12]. The Myelofibrosis Secondary to PV and ET-Prognostic Model (MYSEC-PM) is a prognostic scale specific for patients with sMF that integrates both clinical and molecular features for prognostication [13]. The current benefit of utilizing any of these prognostic models is either in the context of determining clinical trial eligibility or risk-adapted treatment decision making and particularly when considering the role of transplantation [14].

**Table 1:** Prognostication in myelofibrosis includes diverse disease-specific variables that contribute to clinical heterogeneity.

	Lille	IPSS	DIPSS	DIPSSplus	MIPSS70	GIPSS	MYSEC-PM
Age		X	X	X	X		
Leukocytosis	X^a^	X	X	X	X		
Anemia	X	X	X	X	X		X
Symptoms		X	X	X	X		X
Circulating blasts		X	X	X	X		X
Thrombocytopenia				X	X		X
RBC transfusion dependent				X			
Adverse Karyotype^b^				X	X	X	
BMF					X		
Non-CALR type 1					X	X	X
HMR = 1					X^c^	X^d^	
HMR > 1					X^c^		

*IPSS* International Prognostic Scorcing System, *DIPSS* Dynamic International Prognsotic Scoring System, *MIPSS* Mutation-Enhanced International Prognostic Scoring System 70, *GIPSS* Genetically Inspired Prognostic Scoring System, *MYSEC-PM* Myelofibrosis Secondary to PV and ET-Prognostic Model, *RBC* red blood cell, *BMF* bone marrow fibrosis, *HMR* high molecular risk.

^a^Leukocytosis and leukopenia are variables in the Lille prognostic Model.

^b^Complex karyotype or abnormalities including +8, -7/7q-, i(17q), -5/5q-, 12p-, inv(3) or 11q23 rearrangement.

^c^HMR mutations in MIPSS70: *ASXL1*, *EZH2*, *IDH1/2*, *SRSF2.*

^d^HMR mutations in GIPSS: *ASXL1, SRSF2*, *U2AF1*^Q157^.

### Mutational impact on disease phenotype and therapeutic response in myelofibrosis

Of the three driver genes recurrently mutated in MPN, the *JAK2* ^V617F^ mutation is present in 60% of PMF cases, *CALR* mutation accounts for 20-30% of cases with predominance of Type 1/1-like mutation, and *MPL*^W515L/K^ mutation is found in 5-10% of cases. In sMF, *JAK2*^V617F^ is present in almost all cases of PPV-MF and in PET-MF accounts for 50% of them, while *CALR* (Type 1 is the most prevalent) and *MPL* mutation for 30% and 10%, respectively [15]. The *JAK2*^V617F^ variant allele frequency (VAF) ranges from a very low percentage to 100% with a median of approximately 50%; and frequently increases with the transition from PV and ET to sMF, reflecting clonal dominance [16, 17]. Conversely, a low *JAK2*^V617F^ VAF (<25%) is associated with certain features (lower leukocyte count and hemoglobin) of a cytopenic rather than myeloproliferative MF phenotype and represents an independent variable associated with shortened survival in patients with PMF [18]. The absence of any driver mutation is operationally defined as “triple negative” (TN), and accounts for roughly 10% of PMF patients [19]. So called non-canonical *JAK2* and *MPL* mutations may be found in a minority of TN patients by sequencing all gene coding regions by next generation sequencing (NGS) [20]. Triple-negativity is an independent variable for shortened survival in PMF [21]; data in sMF are scant [22].

In addition to the driver mutations, 40–60% of patients with MF compared to <20% of PV and ET [23] harbor deleterious mutations in a variety of myeloid-neoplasm associated genes, including DNA methylation and epigenetic regulators, members of the spliceosome, oncogenes and transcription factors [11, 24–26]. Mutated *ASXL1* is the most common additional genetic abnormality in MF (25–40% of patients) that harbors unfavorable prognostic significance [27–29], and is included among the High Molecular Risk (HMR) mutations [24] together with *EZH2* (4–7%), *IDH1* and *IDH2* (1–3% each), *SRSF2* (8–15%) and *U2AF*1^Q157^ (8–16%) [30]. The presence of any HMR mutation confers shorter OS and LFS to patients with pre-fibrotic and overt PMF, which is compounded by the presence of more than one HMR mutation [2]. Accordingly, HMR and the number of HMR mutations are embedded in the MIPSS70 scores (MIPSS70/plus [11] and v2.0 [31]. On the other hand, the role of *ASXL1* and HMR mutations in general in sMF remains uncertain [26, 32], and the detrimental value of *ASXL1* mutation when it is the only additional mutation has been recently questioned in two independent cohorts of patients [26]. *ASXL1* stands, together with a non-*CALR/MPL* mutated genotype, as the only genetic abnormality that informs survival and non-relapse mortality after stem cell transplantation in MF [14]. Other mutated genes reported at <5% frequency with uncertain impact on survival include *TET2*, *DNMT3A*, *NFE2, SH2B3, CUX1, CBL, RUNX1, NOTCH1, N/KRAS* and *TP53*. Myeloid mutations are enriched in patients with cytopenic versus myeloproliferative MF phenotype [33], including HMR and *U2AF1 gene* mutations (AM Vannucchi et al., 2022, submitted), consistent with prior data indicating clustering with anemia and thrombocytopenia [34].

The frequency of *TP53* mutations, and/or chromosome 7 deletions and/or amplifications of genes encoding negative regulators of p53, such as MDM2, is increased at the time of evolution to secondary acute myeloid leukemia (sAML) [35]. However, a single, stable, low VAF, *TP53* mutation detected at chronic phase has not been unequivocally associated with shorter OS and LFS, suggesting that haploinsufficiency of *TP53* per se may not be sufficient for evolution to sAML [36–38]. Also, the mechanisms by which loss of *TP53* function in association with dysregulated JAK-STAT signaling leads to leukemia remains unclear, but may be related to genetic instability leading to numerous chromosome abnormalities [39]. Of note, some cases of sAML originate from the background of previously *JAK2*^V617F^ –positive hematopoiesis while in other cases blasts are *JAK2* wildtype suggesting *de novo* origin [40]. One important question is whether these latter leukemias originated from an antecedent clonal hematopoiesis of indeterminate potential (CHIP), different from the one that established the chronic phase MPN. Single cell studies reinforce the extreme complexity and heterogeneity of leukemic evolution in MF with multiple subclones branching from the originating leukemic clone [41, 42].

The contribution of mutational profile to the response or resistance to the JAK 1/2 inhibitor ruxolitinib has been thoroughly investigated. In one report, a *JAK2*^V617F^ VAF > 50% was associated with better spleen response to ruxolitinib [43]. Presence of HMR mutations does not significantly impact short-term response to ruxolitinib [44], however it may be associated with a shorter duration of response [45], and acquisition of new mutations configuring clonal progression that contributes to therapy resistance [45–47]. Involvement of the RAS/CBL pathway, that per se predicts shorter OS and LFS [48, 49], may be associated with reduced symptom and spleen response to JAK inhibitors, highlighting the opportunity for dual targeting of JAK-STAT and RAS/MAPK signaling.

### Spectrum of clinical phenotype in myelofibrosis

The heterogeneous nature of MPNs was recognized by Dameshek across the entire spectrum of these diseases, but arguably it is most apparent for patients with MF; in the next sections we explore this theme of clinical heterogeneity within MF particularly focusing upon advanced MF [50].

The mutational landscape of MF has been better delineated in recent years and most non-driver mutations in MF are also prevalent in MDS and AML. Some authors have suggested that certain cases of MF appear not to be a pure MPN, but instead also have features of MDS – including dysplastic morphology and cytopenias etc. Indeed, the presence of non-driver mutations is reportedly correlated with the likelihood of myelodysplastic features and the severity of MF [51]. This highlights the MF disease spectrum ranging from myelodysplastic with consequent cytopenias to myeloproliferative MF. Other authors have focused upon a different terminology and describe advanced MF using the term “myelodepletive MF”. Here the degree of cytopenia associated with the myelodepletive MF phenotype is characterized by severe pancytopenia with low leukocyte count, platelet count, anemia, and frequently requiring transfusions. In contrast, the myeloproliferative MF phenotype is associated with leukocytosis, variable platelet counts, anemia, more frequently massive splenomegaly and a symptom profile associated with abdominal pain and night sweats [33].

These nosological models of myelodysplastic (mutational/morphological profile) and myelodepletive (thrombocytopenia/anemia) MF are likely not mutually exclusive. Whether the preferred term is cytopenic or myelodepletive, which recognizes a distinction from MDS and the WHO entity MDS with fibrosis, we will explore features of this phenotype in more detail below. These phenotypes are not always perfectly represented in individual studies due to the complex pathophysiology driving the clinical heterogeneity of MF, but instead simply represent a framework in which a spectrum of clinical phenotype can be better appreciated. Additionally, the paradox of atypical megakaryocyte hyperplasia which is a pathognomonic bone marrow feature of MF still underlies the thrombocytopenia overrepresented but not exclusive to the cytopenic MF profile. This is in contrast to the myelodepletive concept that may be best suited to convey a pathobiology that more closely resembles myelodysplastic syndrome.

Thrombocytopenia, (platelet count <100 × 10^9^/) a key feature of cytopenic MF, is present in approximately 20% of MF patients at diagnosis with ∼11% presenting with a platelet count of <50 × 10^9^/L and 30% at one year [33]. Thrombocytopenia is a recognized marker of poor prognosis. In the IPSS model, platelet counts <100 × 10^9^/L were associated with decreased survival; however, due to its correlation with anemia, it was discarded as a separate factor [6]. In contrast, the DIPSS-plus [10] and subsequent prognostic scales also retain thrombocytopenia [12, 14]. The MYSEC includes platelets <150 × 10^9^/L and hemoglobin <11 g/dL as poor prognostic markers [13], suggesting that in both PMF and sMF, the degree of thrombocytopenia has independent prognostic impact and serves as a marker of advanced disease.

It is also well established that thrombocytopenic MF patients have less frequent response and a shorter overall duration of response to ruxolitinib [52]. Barosi and colleagues reported that spleen response rates to ruxolitinib were reduced for those with lower rather than a higher *JAK2*^V617F^ VAF. Here ≥ 50% *JAK2*^V617F^ VAF was associated with a 5.5-fold greater probability of a spleen volume response compared with patients with <50% *JAK2*^V617F^ VAF or another mutation [43]. The authors suggest that biology of the disease explains the higher response rate and that targeting JAK2 downstream signaling effectors with ruxolitinib would be more effective in persons with a high *JAK2*^V617F^ VAF.

The previous findings may not however be generalizable across JAK2 inhibitors. Tremblay and colleagues assessed the efficacy of pacritinib, a JAK2/IRAK1 inhibitor, in MF patients with low *JAK2*^V617F^ VAF in a post hoc analysis of the PERSIST-1 and −2 trials [53]. In that study, patients with lower *JAK2*^V617F^ VAF had smaller baseline spleen size and lower hemoglobin and platelet counts as compared to patients with a higher VAF or *JAK2* wildtype MF. Here, pacritinib treatment led to superior spleen and symptom burden reduction compared with BAT in patients with absent/low *JAK2*^V617F^ allele burden, thus, suggesting that pacritinib may be uniquely suited for patients with cytopenic MF.

However, allelic burden is not universally found to correlate with advanced MF and cytopenias, suggesting that the underlying causality is likely more complex. In a cohort of 594 WHO-defined MF patients from Florence, a cytopenic phenotype, defined by ≥1 cytopenia without accompanying cytosis (leukocytes > 15 × 10^9^/L, hemoglobin >16.5 g/dL for male and >16 g/dL for female, platelets >450 × 10^9^/L) was identified in 166 patients [54]. Differences between PMF and sMF were also explored. Cytopenic PMF was associated with male gender (*p* = 0.0468), older age (*p* = 0.0002), lower peripheral blast count (*p* = 0.0006), higher prevalence of splenomegaly (*p* = 0.0142), constitutional symptoms (*p* < 0.0001), and BM fibrosis grade ≥2 (*p* < 0.0001). Also cytopenic MF patients were more likely to have very high-risk karyotypes (*p* = 0.0002), lack a driver mutation (TN; *p* < 0.0001), and also harbor a mutation involving *ASXL1* (*p* = 0.0074), *IDH1/2* (*p* = 0.064), *N/KRAS* (*p* = 0.0014), *U2AF1* (*p* < 0.0001), or *CUX1* (*p* = 0.0002). Karyotypic abnormalities (*p* = 0.0084), very high-risk cytogenetics (*p* = 0.0343), *CBL* (*p* = 0.0.171) and *U2AF1* (*p* = 0.0148) mutations were significantly enriched in cytopenic patients with ≥2 cytopenias. In this study, OS was much lower in cytopenic PMF (median, 55 vs 103 months, respectively; *P* < .0001). Phenotypic differences for cytopenic MF were less evident in sMF, except for older age (*p* = 0.0207), and a molecular landscape which was enriched for mutations in *TP53* (*p* = 0.0024), *U2AF1* (*P* < 0.0001), and *SETBP1* (*p* = 0.0125). Again, cytopenic sMF patients had shorter OS (median, 44 vs 105 months; *p* < 0.0001). Median OS was significantly inferior in those with ≥2 cytopenias compared with one cytopenia (median, 27 vs 58 months, respectively; *p* < 0.0001) [55].

Two large studies identified MF patients with platelets <50 ×10^9^/L in particular as advanced disease. In a multivariable analysis of 1100 MF patients, thrombocytopenia was an independent negative prognostic variable for OS. Patients with platelets <50 ×10^9^/L had other myelodepletive features (lower hemoglobin level, leukocytes, transfusion dependence, circulating blasts, older age, and abnormal/unfavorable karyotype). In this cohort, myelodepletive sMF did not appear to have substantially different clinical characteristics than myelodepletive PMF [56].

A study of the Spanish Registry data compared 57 such patients with 834 patients with a platelet count of ≥50 ×10^9^/L [57]. This severely thrombocytopenic group was more likely to experience additional cytopenias, circulating blasts, MF-3 bone marrow fibrosis, and hemorrhage. Leukemic transformation was more common in the severely thrombocytopenic group (7.0 vs. 2.6 per 100 patient-years; *p* = 0.02), with a median projected survival of 2.2 years. No specific cytogenetic profile was associated with severe thrombocytopenia; however, there was a trend towards a lower frequency of *JAK2*^V617F^, and higher risk IPSS/DIPSS-plus score. Given the short OS, management of MF patients with severe thrombocytopenia constitutes a major unmet clinical need [57].

Additional studies have also demonstrated inferior outcome in PMF patients with low *JAK2*^V617F^ allele burden, however, the relationship to severe thrombocytopenia was not established; Guglielmelli and colleagues stratified 186 PMF patients into: 1% to 25%, 25% to 50%, 50% to 75% and >75% *JAK2*^V617F^ VAF [18]. The lowest quartile developed anemia and leukopenia more rapidly, but not particularly thrombocytopenia. In a prognostic model based on mutation status using an MD Anderson cohort of 344 PMF patients, a 50% cut-off dichotomized *JAK2*^V617F^ patients into those with high *JAK2*^V617F^ VAF and favorable survival and those with low *JAK2*^V617F^ VAF and unfavorable survival [58].

### Role of inflammatory signaling in myelofibrosis

In healthy individuals, inflammation is driven by a delicate interplay between cellular responses and stimulatory factors. Dysregulation of this inflammatory cascade is a hallmark of MPNs and the chronic inflammatory state of MF in particular, that is implicated in the debilitating constitutional symptoms and cytopenias characteristic of the disease [59, 60]. In Philadelphia chromosome-negative MPNs, driver mutations converge upon the JAK2/STAT3/STAT5 pathway [51], leading to its constitutive activation that can drive cytokine hypersensitivity, myeloid and megakaryocyte proliferation and differentiation [61]. NFκ-B remains the central transcriptional regulator of a wide array of inflammatory cytokines aberrantly expressed in MF that include interleukin (IL)−6, IL-1β, IL-8, tumor necrosis factor (TNF)-α, transforming growth factor beta (TGF)β and many others [62, 63]. NFκ-B is activated downstream of Toll-like receptors (TLR) whose signaling is upregulated in both malignant and non-malignant stromal cells of MF [64]. A constitutively activated JAK/STAT pathway can also drive sustained NFκ-B activation. The *JAK2*^V617F^ gain of function mutation promotes p53 degradation through the accumulation of the E3 ubiquitin ligase HDM2 (Human Double Minute 2) *via* the La translational promoter in MPNs [65]. As a result, HDM2 increases NFκB activity by directly binding the Sp1 promoter site of NFκB p65 to activate its transcription [66]. Importantly, aberrant JAK2 signaling in MF and other MPNs leads to epigenetic changes that can also enhance NF-kB signaling [67].

**Fig. 1:**
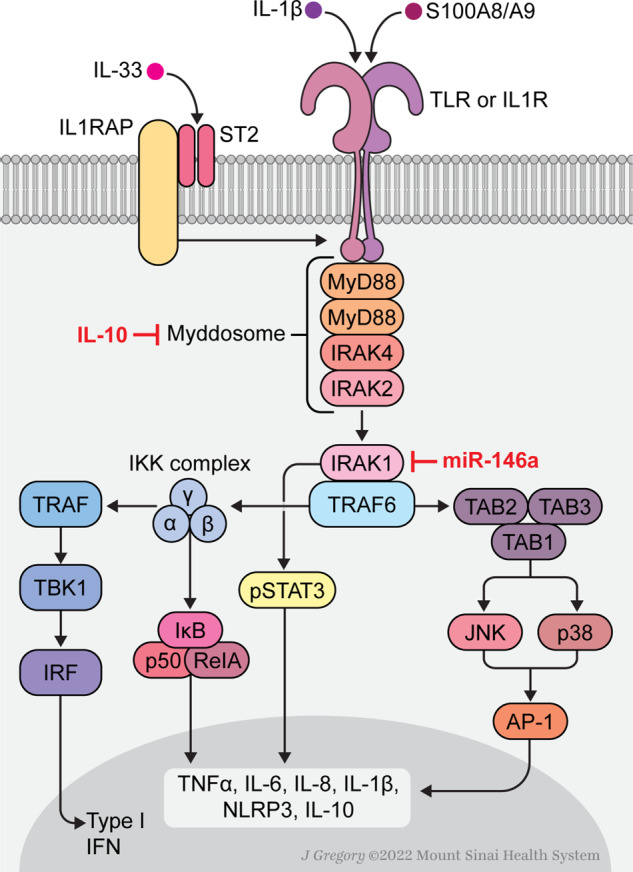
Myddosome signaling. Ligand engagement of TIR -domain containing receptors triggers their dimerization and myddosome assembly through recruitment of the TIR-domain containing adaptor protein, MyD88. The N-terminal death domains (DD) of MyD88 proteins interact with the DD-containing, serine/threonine IRAK family kinases to create the active macromolecular protein signaling complex that converges upon IRAK1 transphosphorylation. Phosphorylated IRAK1 dissociates from the myddosome complex to activate the E3-ubiquitin ligase TRAF6 that is responsible for the activation of several transcription factors including NF-kB, AP-1, and interferon (IFN) regulatory factors (IRFs). Collectively, these proteins induce the expression of pro-inflammatory cytokines, IFNs and IFN-stimulated genes (ISGs), components of the NLRP3 inflammasome as well as the anti-inflammatory cytokine IL-10, which serves to quench myddosome signaling. The microRNA, miR-146a, also suppresses myddosome signaling through degradation of *IRAK-1, TRAF-6* and *TGF-β* gene transcripts. IRAK1 also directly phosphorylates STAT3, triggering its nuclear translocation independently of the Janus kinases.

The transmembrane TLRs contribute to this initial inflammatory signal, as monocytes from MPN patients are hyper-responsive to TLR ligands, which directs excess inflammatory cytokine production [68] (Fig. 1). TLRs, together with the IL-1 receptors, are part of a superfamily of pattern recognition receptors essential to the innate immune response that recognize pathogen-associated molecular patterns (PAMPs) from various microbes and self-derived molecules from damaged cells, known as damage-associated molecular patterns (DAMPs) that are over-expressed in both MPN patients and murine models [59, 64, 69]. Signaling downstream of TLRs is initiated by conformational changes induced by ligand-binding that leads to the recruitment of Toll/IL-1 receptor (TIR) domain-containing adaptor proteins that bind to the corresponding cytoplasmic TIR regions of TLRs [70, 71]. Five TLR adaptor proteins are known to interact, specifically Myeloid differentiation factor 88 (MyD88), TIR-domain containing adaptor molecule (TRIF), MyD88-adaptor-like (MAL, also known as TIR domain containing adaptor protein [TIRAP]), TRIF-related adaptor molecule (TRAM), and sterile α- and armadillo-motif-containing protein (SARM) [72]. MyD88-driven signaling primarily leads to the production of inflammatory cytokines such as TNF, IL-6, IL-1 and chemokines (CCL4), whereas TRIF induces the expression of type I and type II IFNs [71].

### Myddosome deregulation

Two large signaling complexes referred to as supramolecular organizing centers or SMOCs direct innate immune response to TLR activation, namely the TLR-associated *myddosome* and the cytosolic, *inflammasome* complexes [73]. After TLR activation by PAMPs or DAMPs, the MyD88 adaptor rapidly initiates assembly of the myddosome complex via association with its death domain [70]. Key effectors include the serine/threonine kinase interleukin-1 receptor-associated kinase (IRAK)4, which phosphorylates its homolog IRAK1, triggering its auto-phosphorylation and dissociation from the myddosome, whereby it associates with the TNF receptor-associated factor (TRAF)-6. Poly-ubiquitination by TRAF6 activates IRAK1 signaling and the downstream induction of a large group of inflammatory cytokines through the activation of a variety of transcription factors including NF-κB, interferon regulatory factor (IRF)5, activator protein 1 (AP-1), and cAMP response element binding protein (CREB), as well as the activation of c-Jun N-terminal kinase (JNK) and p38 mitogen-activated protein kinase (MAPK). IL-10 is the only anti-inflammatory cytokine generated by the IRAK1/NF-kB axis that serves as a negative feedback loop to extinguish myddosome-signaling [74]. IRAK1 also directly phosphorylates the transcription factor STAT3, triggering its nuclear translocation independent of the Janus kinases. Upon joint nuclear translocation with STAT3, IRAK1 phosphorylates Histone H3 thereby enhancing promoter binding by NF-kB to up-regulate inflammatory cytokines [75]. Cytokines under transcriptional control of NF-kB (e.g., TNFα, IL-6 & IL-8) are not extinguished by JAK1 or JAK2 inhibition in MF; however, they are suppressed by IRAK1 inhibition accompanied by a reduction in CD34 + colony formation [67, 76]. The activation of IRFs results in the transcription of a host of interferon-stimulated genes through the engagement of JAK1-associated interferon receptors [77].

A recent study by Muto et al. showed that TRAF6 can act as a tumor repressor and that loss of TRAF6 in pre-leukemic cells, which is associated with MYC-dependent signals, leads to overt myeloid leukemia [78]. Critically, the repression of TRAF6 has been observed in a subset of patients with myeloid malignancy, suggesting that dysregulation of TRAF6 can lead to acute leukemia. Moreover, miR-146a, which targets TRAF6 and IRAK1 mRNA for degradation, was shown to act as a tumor suppressor in the hematopoietic compartment and can control myeloproliferation in the spleen and BM through negative regulation of NF-kB [79], hence further linking chronic inflammation through MyD88 in MPNs. Importantly, somatic mutations of *U2AF1* that occur in 10–15% of patients with PMF cause alternate splicing of *IRAK4* gene transcripts to yield a longer isoform retaining exon 4, encoding a protein, IRAK4-Long (L) that is oncogenic in myeloid malignancies and can alone drive proliferation of the malignant clone through sustained myddosome activation [80].

Many studies suggest that inflammation supports MPN pathogenesis and development, yet it remains unclear whether inflammation is an event that initiates myeloproliferation and disease development, or simply a consequence or “byproduct” of the disease. For example, the hyper-responsiveness of monocytes and other hematopoietic progenitors to TLR ligands in MF relates in part to myddosome resistance to physiologic quenching by IL-10 [81]. Moreover, excess generation of TNFα paradoxically fosters the clonal expansion of *JAK2*^*V617F*^ hematopoietic stem and progenitor cells (HSPC) while suppressing the growth of normal progenitors, indicating that the inflammatory bone marrow microenvironment in MF is conducive to propagate *JAK2*^*V617F*^-positive clones [82]. Interestingly, TLR4-directed myddosome signaling in CD34^+^ progenitors from patients with MF induces overexpression of the micro-RNA (miR)155, which degrades *Jumonji And AT-Rich Interaction Domain Containing* 2 (*JARID2)* gene transcripts giving rise to megakaryocytic hyperplasia that is characteristic of the disease [83]. More importantly, Rahman et. al. recently showed using gene silencing and cytokine neutralization approaches that IL-1 receptor/myddosome signaling is indispensable for clonal expansion, megakaryocyte proliferation and progression of MF in a *JAK2*^*V617F*^ knock-in mouse model [84]. These findings were confirmed in a separate study by Rai et. al. which, in addition, demonstrated that serum IL-1β was derived from *JAK2*^*V617*^-mutant HSPC while HSPC IL-1 receptor expression and serum cytokine concentration directly correlated with *JAK2*^*V617F*^ mutant allele fraction [85]. Moreover, antibody neutralization of IL-1β in a *JAK2*^*V617*^-mutant murine model reduced myelofibrosis and osteosclerosis, which was additive to the effects of ruxolitinib, suggesting that strategies that effectively mitigate IL-1 receptor signaling could be disease modifying.

Recent investigations implicate deregulation of *miR-146a* in the constitutive activation of myddosome signaling and peripheral blood cytopenias of MF. *miR-146a* targets *IRAK-1, TRAF-6* and *TGFβ* gene transcripts for degradation and its expression is down-regulated in peripheral blood granulocytes of MF patients [86, 87]. De-repression of these genes in *miR-146a* knock-out mice results in sustained STAT3 activity associated with development of extensive medullary fibrosis, megakaryocytic hyperplasia, anemia, thrombocytopenia and splenomegaly, features that closely phenocopy cytopenic MF [79]. In addition, upregulation of the macrophage colony-stimulating factor receptor (CSF1R) was found in the myeloid cell population. These findings were reversed by selective knock-down of *NF-κB p50*, confirming the critical role of constitutive myddosome signaling in the pathogenesis of cytopenic MF. The precise pathobiology underlying the impairment of hematopoiesis may relate in part to the downstream effects of interferon gamma (IFNγ) induction [88]. IFNγ receptor stimulation leads to release of the alarmin, high mobility group box-1 protein (HMGB1), which disrupts the bone marrow endothelial niche, while deletion of *IFNγ* prevents HMGB1 release and is sufficient to reverse the endothelial defect and restore myelopoiesis [89]. HMGB1 was also recently identified as a key mediator of the anemia of inflammation by physically displacing the binding of erythropoietin to its cognate receptor [90]. As a result, HMGB1 reduces the proliferation and increases cell death of erythroid precursors to exacerbate anemia. Additionally, sustained STAT3 activation upregulates transcription of the *GLI1* gene in MF fibrocytes to activate pro-fibrotic pathways in fibrocyte progenitors [91]. Interestingly, a polymorphism in the *miR-146a* gene, rs2431697, was identified in MPN patients at higher risk for progression to sMF [86]. In a large cohort analysis, the rs2431697 TT genotype was found to be an independent co-variate for higher risk of progression to sMF, findings that were confirmed in a separate cohort analysis of PV and ET patients [92]. Importantly, patients with the rs2431697 TT genotype in both studies had significantly higher levels of plasma inflammatory cytokines.

The second key innate immune SMOC, i.e., the inflammasome, is emerging in importance in the pathobiology of MF. Inflammasome complexes initiate caspase-1 mediated maturation of IL-1β and IL-18 as well as an inflammatory, lytic cell death termed pyroptosis [93]. Among the cytosolic NOD-like receptors (NLRs) that serve as sensors for specific inflammasomes, the pyrin domain containing 3 (NLRP3) inflammasome is the most studied and is critical in both sterile and non-sterile inflammation. The NLRP3 inflammasome, which is abundant in myeloid cells, is composed of the intracellular sensor NLRP3, the adaptor apoptosis-associated speck-like protein containing a caspase-recruitment domain (ASC), pro-caspase-1 and finally its substrates pro-IL-1β, -IL-18 and the pyroptosis executioner, gasdermin-D [93, 94].

Upon NLRP3 inflammasome activation, pro-caspase-1 undergoes auto-catalytic cleavage to functional caspase-1, which in turn transforms pro-IL-1β and pro-IL-18 into their mature, active forms that are subsequently released from the cell [95]. NLRP3 activation is particularly interesting, as it requires two stimuli for activation. The first or “priming” signal is mediated by TLRs in response to PAMPs, stress-associated signals released from DAMPs or IL-1 receptor engagement, or alternatively, after stimulation by TNF-α or IL-6, which have been implicated in priming of the NLRP3 inflammasome with advancing age [68, 96, 97]. This results in transcriptional upregulation of each of the necessary inflammasome components via NF-κB. The functional activation of the NLRP3 inflammasome is mediated by “signal 2”, which can take the form of exogenous or endogenous PAMPs and DAMPs. DAMPs, which are released after cell damage, can include extracellular adenosine triphosphate (eATP), mitochondrial reactive oxygen species (ROS), HMGB1, calcium-modulated proteins including S100A9 and S100A8, uric acid crystals, and extracellular DNA and RNA fragments. This is particularly relevant as various DAMPs are up-regulated and have been implicated in the pathobiology of MPNs, such as S100A9 and IL-33 amongst others [64, 69].

Recent data from Zhou et al. showed that inflammasome-related genes, such as *NLRP3*, are highly expressed in the bone marrow of MPN patients and that increased expression was associated with *JAK2*^*V617F*^, leukocytosis, and splenomegaly [98]. A second inflammasome that is relevant in MPN is the Absence In Melanoma 2 (AIM2) inflammasome that recognizes double-stranded DNA, functioning to protect against pathogens and dsDNA released from apoptotic or dying cells, which in turn leads to the release of proinflammatory cytokines and sterile inflammation [99]. In vitro work using the D9 cell line showed that Aim2 inflammasome-related genes, such as *AIM2*, *CASP1* and *IL1*β, are upregulated upon induction of *JAK2*^*V617F*^, further linking MPN and inflammasome activation [100]. Although the role of the NLRP3 inflammasome is well described in MDS, its role in MPN pathogenesis remains understudied [101].

### DAMPS and the S100A8/S100A9 heterodimer

**Fig. 2:**
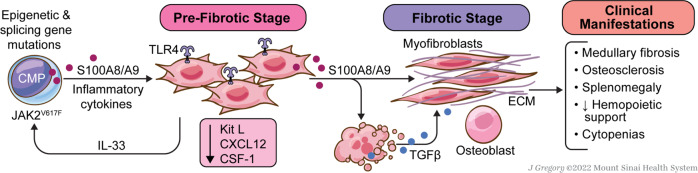
TLR-directed pathogenesis of cytopenic myelofibrosis (MF). *JAK2V617F-*mutant HSPC overexpress the heterodimeric alarmin, S100A8/S100A9, also termed calprotectin. Paracrine stimulation of TLR4 in bone marrow mesenchymal stromal cells (MSCs) instructs aberrant upregulation by MSCs. Calprotectin from MSCs stimulates the TLR4/Myddosome pathway in megakaryocytes to elaborate TGF-β and drive fibrosis progression, splenomegaly and exacerbation of cytopenias in MF.

Recent studies have shown that the alarmin protein heterodimeric tetramer S100A8/S100A9 or calprotectin, which is primarily expressed in monocytes and granulocytes, is significantly upregulated in both murine models of MF and human stromal cells, i.e., cell populations that normally do not express S100A8/S100A9 at steady state [64] (Fig. 2). Critically, S100A8/S100A9 expression in mesenchymal stromal cells (MSCs) occurred with disease progression in both murine models and patient stroma, indicating that this may serve as an advanced disease biomarker. Indeed, calprotectin from MSCs stimulated the TLR4/Myddosome pathway in megakaryocytes to elaborate TGF-β and drive fibrosis progression and splenomegaly in MF patients. Most importantly, the S100A8/S100A9 heterodimer can be targeted in MPN murine models using the small molecule inhibitor tasquinimod, which significantly ameliorated the MF phenotype by reducing bone marrow fibrosis accompanied by spleen size reduction in *JAK2*^*V617*F^-driven models. As inhibition of alarmins in MDS can ameliorate the pathognomonic anemia through inhibition of an inflammatory cascade, it can be assumed that inhibition of alarmins in MF can also have a positive effect on debilitating cytopenias [101].

Cytopenias, and anemia in particular, can be exacerbated by high circulating levels of calprotectin. Calprotectin sequesters cationic transition metals (e.g., calcium, iron, and zinc) that underlies its antimicrobial properties, while Fe^+2^ chelation restricts availability to the erythron, thereby contributing to iron-restricted anemia [102]. Moreover, the inflammatory proteins S100A9, TNFα and IL-1β each suppress transcription and cellular elaboration of erythropoietin, thereby reducing its availability to and stimulation of the erythron [103]. Molecular and proteomic analyses of CD34^+^ progenitors and granulocytes from patients with MPNs have shown that S100A8 and S100A9 are profoundly overexpressed in MF, with plasma levels that exert proliferative signals via the TLR4 and RAGE receptors, unrelated to *JAK2*^*V617F*^ status [104, 105]. Of interest, plasma concentrations of S100A8 and S100A9 were highest in those patients with lower *JAK2*^*V617F*^ VAF, a molecular feature more common in patients with cytopenic MF. Increased calprotectin production in cytopenic MF arises in part from the traditional non-driver, somatic gene mutations common to this disease subset. Although unrestrained JAK2/STAT activation induces S100A8/S100A9 in clonal hematopoietic progenitors and the surrounding stroma, both epigenetic regulatory and mRNA spicing gene mutations induce S100A8 and S100A9 overexpression to compound ineffective blood production in cytopenic MF [64, 106, 107]. Importantly, overexpression of *S100A9* in a transgenic mouse model is alone sufficient to cause pancytopenia as a result of ineffective hematopoiesis that was ameliorated by NLRP3 inflammasome inhibition [108]. S100A9 also directs ineffective hematopoiesis by inducing the expression of the PD-1 death receptor on HSPC and its corresponding ligand, PD-L1, on myeloid-derived suppressor cells (MDSCs) [109].

### Interleukin- 33

Interleukin-33 (IL-33), a member of the IL-1 cytokine family, signals via the myddosome analogous to its TLR family members. Full-length IL-33 is biologically active and is primarily expressed by epithelial and endothelial cells, however, IL-33 may exert a dual function in that it acts as an “alarmin” (DAMP) extracellularly, or as a nuclear factor modulating gene expression [110, 111]. Increased levels of IL-33 are demonstrable in the bone marrow and splenic vascular endothelium of MPN patients compared to controls, similar to expression of its accessory receptor ST2, also known as IL1RL1, which is necessary for IL-33 binding to the receptor complex and is upregulated in CD34 + HSPCs [69]. Binding of IL-33 to its cognate ST2 receptor initiates complex formation with the IL-1 receptor via the IL-1R accessory protein (IL-1RAcP) subunit. Signaling by the heterodimeric receptor is thereby mediated through the myddosome [112]. Mager et al. showed that IL-33 is an important contributor to the development of *JAK2*^*V617F*^-driven MPN in mice and exogenous IL-33 promotes colony formation of human primary CD34 + MPN HSPCs [69]. Interestingly, the IL-33/ST2 pathway can be activated in both the hematopoietic compartment and in non-hematopoietic MSC. IL-33 also stimulates the secretion of other DAMPs such as S100A8/S100A9 [113], which are strongly implicated in directing the MF phenotype [64].

### Non-driver mutations and association with the cytopenic MF profile

The nuclear factor erythroid-2 gene (*NF-E2*), a hematopoietic transcription factor, is critical for proper differentiation of erythroblasts and megakaryocytes [114]. A study of 2,035 MPN patients (PMF 184; PV 411; ET 577) showed that the cohort harboring *NF-E2* mutations frequently was *JAK2* ^*V*617F^ homozygous, and *NF-E2* mutations were acquired significantly later in the disease course [25]. Another study also showed that mutations in *NF-E2* were detected in MPN patients who harbored *JAK2* ^V617F^, provided a proliferative advantage to the doubly mutant clone; and in a murine model, *NF-E2* mutations caused a myeloproliferative phenotype (erythrocytosis and thrombocytosis) while predisposing to leukemic transformation [115]. Guglielmelli et al. reported that *NF-E2* mutations were twofold more frequent among 631 MPN patients who had *JAK2* VAF > 50%, however, there were no clear prognostic or meaningful clinical/hematological correlates [116].

Conversely, in a larger recent study, multivariate analysis of the data from 707 patients with MPNs (113 PMF, 233 PV, 332 ET) and available NGS data demonstrated that *NF-E2* mutations (VAF ≥ 5%) carried a hazard ratio (HR) of 10.29 for transformation to AML (independently from age and co-occurring HMR mutations) and an HR of 8.24 for OS [117]. The HR of *NF-E2* mutations was about fivefold higher for leukemogenesis and fourfold higher for OS compared to HMR mutations, respectively, thereby associating *NF-E2* gene mutations with an aggressive disease course [117]. Notably, the *NF-E2* VAF decreased at the time of leukemic transformation compared to the chronic phase, indicating that *NF-E2*-mutated cells may be outcompeted by another new dominant clone. In this case, *NF-E2* acts as a “sentinel” mutation, dramatically increasing the likelihood of acquiring other mutations and leukemogenesis via a paracrine effect [118].

Patients harboring *NF-E2* mutations had a higher median hematocrit than non-mutated patients in line with its association with the myeloproliferative phenotype and higher incidence in PV (7.3%) vs. PMF (5.3%) and ET (3.6%) [117]. As previously noted, *NF-E2* mutations were acquired later in the disease course [25], induced significantly lower rates of hematological responses leading to the necessity for more lines of treatment, and were detected in 40% of the patients who lost response to treatment [117]. On the basis of these findings, analysis of *NF-E2* mutations can be performed at diagnosis and in follow-up or upon loss of response to treatment. In patients harboring *NF-E2* mutations, histone deacetylase inhibitors may be a rational therapeutic given their downregulation of *NF-E2* expression [119].

Spliceosome *U2AF1* mutations are detected in 10–15% of PMF patients [51], and are associated with the cytopenic MF phenotype and with ≥2 cytopenias [54]. In addition, *U2AF1*^Q157^ was associated with inferior survival compared to wild type-*U2AF1* [30]. For this reason, *U2AF1*^Q157^ is included with other HMR mutations in the MIPSS70-plus v.2.0 and GIPSS prognostic models for PMF [120, 121]. Tefferi et al. noted a phenotypic correlation of the spliceosome pathway mutations *U2AF1* and *SRSF2* with anemia [122]. In PMF, both *U2AF1*^Q157^ and *U2AF1*^S34^ mutations were strongly associated with severe anemia (Hg < 10 g/dL); and *U2AF1*^Q157^ specifically with thrombocytopenia (platelet count <100×10^9^/L) [30, 34]. The strong association of mutant *U2AF1* with anemia and thrombocytopenia was sustained when mutated-*JAK2* and wild type *JAK2* patients were analyzed independently; *U2AF1* mutations directly associated with *JAK2*^V617F^ [34].

## Summary

MF is a hematopoietic stem and progenitor cell-derived malignancy with complex molecular underpinnings and an associated immune deregulated state. The phenotypic spectrum ranges from the cytopenic to proliferative MF clinical and hematological profile that relates in part to somatic JAK-STAT driver mutations, the presence of accompanying non-driver mutations, and aberrant cell-intrinsic and -extrinsic inflammatory pathways. The cytopenic MF patient population is enriched for the PMF subtype, clinically typified by cytopenias and less extensive splenomegaly, and molecularly characterized by wild-type *JAK2* or low *JAK2*^V617F^ VAF frequently accompanied by somatic mutations involving the spliceosome, epigenome, and apoptotic pathways. Due to inferior OS, higher risk of leukemic transformation and increased resistance to ruxolitinib therapy, cytopenic MF patients pose a therapeutic challenge and represent an unmet medical need. Recognition of the role of TLR signaling and downstream myddosome activation of NFκB mediated pro-inflammatory cytokine expression has provided novel therapeutic targets for MF, such as IRAK1. Tailoring treatment with JAK inhibitors to genotype and phenotype will extend the potential for clinical benefit across the disease spectrum.
